# Failed Attempts at Oxidation of XeF_6_: Synthesis and Characterization of [Xe_2_F_11_][RuF_6_]

**DOI:** 10.1021/acs.inorgchem.6c02072

**Published:** 2026-06-24

**Authors:** Björn N. Koch, Giuliana Hoß, Gregor Schnakenburg, Antti J. Karttunen, Florian Kraus

**Affiliations:** † Fluorchemie, Institut für Anorganische Chemie, 9374 Universität Bonn, Gerhard-Domagk-Strasse 1, 53121 Bonn, Germany; ‡ Department of Chemistry and Materials Science, 174277 Aalto University, Kemistintie 1, Aalto Finland 00076

## Abstract

Herein we report our attempts to oxidize XeF_6_ using RuF_6_ in anhydrous HF (aHF) under various conditions. Contrary to expectations of forming a Xe­(VIII) species such as [XeF_7_]^+^, the reactions consistently resulted in the formation of F_2_ gas and, depending on the stoichiometric ratio, either the known compound [XeF_5_]­[RuF_6_] or the novel Xe­(VI) compound [Xe_2_F_11_]­[RuF_6_]. The latter was characterized by single-crystal X-ray diffraction and vibrational spectroscopy and studied with quantum chemical calculations. These, together with the consideration of reaction and lattice energies, suggest that an ionic solid containing [XeF_7_]^+^ cations may be stable. However, we did not observe its formation under our reaction conditions, as the decomposition of [XeF_7_]^+^ cations may be too fast in our case.

## Graphical abstract

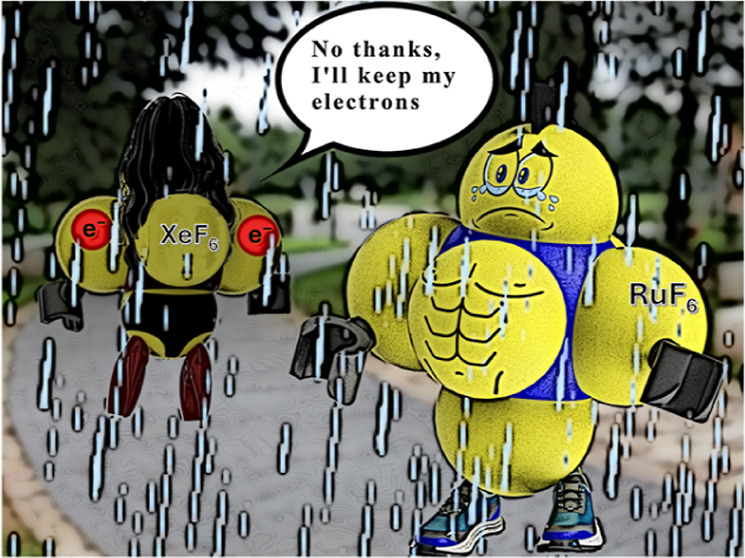

## Introduction

The first noble gas compound was discovered by Neil Bartlett in 1962. The reaction of PtF_6_ with Xe resulted in a yellow reaction product, which was proposed to be “XePtF_6_”.^1^ Subsequent studies revealed that the composition was in fact a mixture of various [XeF]^+^ salts.^2^ This discovery contradicted the prevailing view at the time that noble gases were inert. Furthermore, it paved the way for the development of a very rich noble gas chemistry in the following decades.^3–12^ Currently, the binary noble gas fluorides KrF_2_, XeF_2_, XeF_4_, XeF_6_, and likely RnF_2_ are known.^13–16^ Due to the short half-life of Rn, RnF_2_ is not well characterized.^3,17^

Although the existence of XeF_8_ was suggested by Slivnik and co-workers,^18^ various attempts on synthesizing this compound through the reaction of F_2_ with Xe failed.^19^ In addition, it is predicted to be thermodynamically unstable with respect to loss of F_2_.^20^ Besides using F_2_ as the oxidizer, various other attempts were made to synthesize a perfluorinated Xe­(VIII) compound. For example, XeF_6_ was reacted with the two strongest fluorinating agents known so far, [KrF]^+^ and [NiF_3_]^+^.^21,22^ However, XeF_6_ is a decent fluoride ion donor and the reaction is leading to [XeF_5_]^+^ and the corresponding neutral or anionic species of the oxidizing agent, as shown in eqs 1 and 2. Both KrF_2_ and [NiF_6_]^2–^ are far less potent as an oxidizer.^6,21^

1 $${\left[KrF\right]}^{+}+{XeF}_{6}\to {KrF}_{2}+{\left[{XeF}_{5}\right]}^{+}$$

2 $${\left[{NiF}_{3}\right]}^{+}+3{XeF}_{6}\to {\left[{NiF}_{6}\right]}^{2-}+3{\left[{XeF}_{5}\right]}^{+}$$

Our latest research has demonstrated that, in addition to [KrF]^+^ and [NiF_3_]^+^, RuF_6_ is the only oxidizing agent that is capable of oxidizing BrF_5_ to [BrF_6_]^+^.^23^ Furthermore, the RuF_6_ molecule exhibits far less Lewis acidity compared to the cationic species [KrF]^+^ and [NiF_3_]^+^.^24^ Consequently, RuF_6_ should be a more suitable reagent for oxidizing strong Lewis bases. For RhF_6_, only very few reactions have been carried out, and it essentially cannot be distinguished from RuF_6_ in oxidizing ability. Since RhF_6_ has been reported to be even more thermally unstable than RuF_6_, it might be expected to be a somewhat stronger oxidant.^25,26^ However, according to calculations, RhF_6_ is a weaker oxidizing agent with an electron affinity of 6.8 eV compared to RuF_6_ (7.0 eV).^24^

Due to its high ionization energy, the noble gas Xe only reacts with F_2_ or strong fluorinating agents such as PtF_6_.^27^ Nevertheless, compounds such as oxides and oxyfluorides can be formed through the hydrolysis of Xe fluorides. In contrast to thermodynamically stable fluorides, these oxides are endothermic and tend to explosive decomposition at standard conditions.^28–31^ Nevertheless, XeO_3_ undergoes disproportionation in an acidic solution, forming the often highly unstable perxenates­(VIII) [XeO_6_]^4–^, with the exception of the Na and Ba salts having decomposition temperatures of ca. 360 and 300 °C,^32^ respectively, and also XeO_4_.^33^ Apart from these compounds, XeO_3_F_2_ is the only one compound known to contain Xe­(VIII) atoms. However, XeO_3_F_2_ is so unstable that it can only be matrix-isolated.^30,34^

## Results and Discussion

For the attempted synthesis of a Xe­(VIII) compound, such as XeF_8_ or a [XeF_7_]^+^ salt, RuF_6_ and XeF_6_ were reacted in aHF at different ratios and temperatures. The decolorization during reduction of the intensive brown RuF_6_ provided good first evidence for a complete reaction, especially when XeF_6_ was used in excess. Since RuF_6_ is a one-electron oxidizing agent, a 2:1 ratio was supposed for a complete reaction according to eq 3.

$$“2{RuF}_{6}+{XeF}_{6}\to \left[{XeF}_{7}\right][{Ru}_{2}F_{11}]”$$ 3

Both starting materials are a little volatile, with 89 mbar at 17.6 °C for RuF_6_ and 38.5 mbar at 25 °C for XeF_6_.^35,36^ Therefore, it can be quite challenging to meet the desired stoichiometric ratio exactly. RuF_6_, XeF_6_, and aHF were condensed together at liquid N_2_ temperature. After thawing, the brown RuF_6_ immediately decolorized when the HF melted. A yellowish solution was obtained that evolved gas. The solution was cooled to −196 °C and the remaining gas phase was pumped through KI powder, resulting in a color change from colorless to brown showing the formation of I_2_. Since the other remaining fluorination agents are not volatile at −196 °C, this was an unambiguous indication for the formation of F_2_, which has a vapor pressure of ca. 370 mbar at this temperature.^37–40^ The remaining volatile compounds were pumped off at room temperature. However, the reaction product gave no evidence for the formation of a species containing Xe­(VIII) atoms; instead the Xe­(VI) compound [Xe_2_F_11_]­[RuF_6_] was formed, according to eq 4, as evidenced by single crystal and powder X-ray diffraction and vibrational spectroscopy. The compound also forms without aHF as a solvent. Its characterization is given below.

$${RuF}_{6}+2{XeF}_{6}\overset{aHF\;\text{or}}{\underset{\begin{array}{c} neat \end{array}}{\to }}\left[{Xe}_{2}F_{11}\right]\left[{RuF}_{6}\right]+\frac{1}{2}F_{2}$$ 4

When RuF_6_ and XeF_6_ were reacted in an equimolar ratio in aHF as a solvent, the formation of F_2_ was also observed. In addition, a colorless solid immediately precipitated at room temperature. After the solvent was pumped off at room temperature, the known Xe­(VI) compound [XeF_5_]­[RuF_6_] was obtained,^41,42^ according to eq 5. Its powder X-ray diffraction pattern and Raman spectrum are given in the Supporting Information.

5 $${RuF}_{6}+{XeF}_{6}\overset{aHF}{\to }\left[{XeF}_{5}\right]\left[{RuF}_{6}\right]+\frac{1}{2}F_{2}$$

When a 2:1 ratio of RuF_6_ and XeF_6_ was used or when a larger excess of RuF_6_ was present in aHF, F_2_ gas formed after thawing. After removal of the volatiles in vacuum, a dark-green oily liquid, presumably of RuF_5_, formed. Equation 6, which is hypothetical as we could not quantify the formed products, describes the reaction. When cooled to dry ice temperatures to record a powder X-ray pattern, the dark-green solid of RuF_5_ turned out to be X-ray amorphous. This behavior of RuF_5_ is in principle in accordance with our experiences with MoF_5_.^43^ Raman spectroscopy showed bands assignable to [XeF_5_]^+^ cations, RuF_5_, and (oligo)­fluoridoruthenate­(V) anions [Ru_*n*_F_5*n*+1_]^−^ (*n* = 1, 2, 3, ...). The Raman bands of such [Ru_*n*_F_5*n*+1_]^−^ anions overlap and no clear assignment to single species is possible. However, no evidence for [XeF_7_]^+^ cations was present, of which the most intense Raman bands were quantum-chemically predicted at 588, 647, and 503 cm^–1^.^20^

$$m+n{RuF}_{6}+{XeF}_{6}\overset{aHF}{\to }\left[{XeF}_{5}\right]\left[{Ru}_{n}F_{5n+1}\right]+\frac{m+n}{2}F_{2}+m{RuF}_{5}$$ 6

Highly oxidized fluorides can often be stabilized at lower temperatures. Nevertheless, when the starting materials are reacted at −78 °C in aHF the decolorization of RuF_6_ is much slower. However, the formation of F_2_ gas is also observed. Immediately after the intensive brown color of RuF_6_ disappeared the formation of F_2_ gas stopped. Also, in this case only [XeF_5_]^+^ salts were observed in the Raman spectra. The products resulting from the reactions at −78 °C were found to be equivalent to those obtained at room temperature. As previously outlined, the formation of these products depends on the stoichiometric ratio of RuF_6_ and XeF_6_.

### Why does RuF_6_ fail to oxidize XeF_6_ (at least not under these conditions)?

Of course, we can only outline our thoughts in relation to the reaction conditions used in our study.

### The choice of solvent aHF and comparison of the reactivity of oxidizers:

Anhydrous HF is a very redox-stable solvent, as even compounds with higher oxidative power than F_2_, such as, e.g., PtF_6_, RuF_6_, Ni­(IV), and Ag­(III) salts, can be handled in it.^44^

[KrF]^+^ and [Kr_2_F_3_]^+^ are able to oxidize O_2_ to [O_2_]^+^, ClF_5_ to [ClF_6_]^+^, BrF_5_ to [BrF_6_]^+^, IF_5_ to [IF_6_]^+^, Xe to [XeF_5_]^+^, and Au to AuF_5_ and [AuF_6_]^−^.^5^ However, as stated in the introduction, their Lewis acidity renders them unsuitable for oxidizing fluoride ion donors like XeF_6_.

Ni­(IV) and Ag­(III) dissolved in aHF have been described by Bartlett and others as potentially the most powerful oxidizers since these convert solutions of Pt­(V) or Ru­(V) salts in aHF to the respective hexafluoride.^44–46^ Similar to [KrF]^+^ and [Kr_2_F_3_]^+^ these compounds are also Lewis acids. So, the reaction of NiF_4_ or [NiF_3_]^+^, respectively, with XeF_6_ in aHF at −65 °C led only to the formation of [XeF_5_]_2_[NiF_6_]. Using a large excess of XeF_6_, [Xe_2_F_11_]_2_[NiF_6_] was obtained.^44^ The latter is also formed from NiF_2_, KrF_2_, and XeF_6_ in aHF.^47^ In both cases, no evolution of F_2_ was observed in contrast to our reaction with RuF_6_. For Ag­(III) compounds a similar outcome is possible, forming [AgF_4_]^−^ salts, but to our knowledge this has not been investigated so far.

PtF_6_ does oxidize ClF_5_ but yields a mixture of [ClF_6_]^+^ and [ClF_4_]^+^.^48,49^ PtF_6_ does not oxidize BrF_5_.^50^ However, RuF_6_ gives a clean reaction with ClF_5_ to [ClF_6_]^+^.^23^ It oxidizes BrF_5_ to [BrF_6_]^+^ and also converts lower Pt fluorides to PtF_6_,^23,51^ rendering it a stronger oxidant than PtF_6_. For the reactivity of the other metal hexafluorides, the reader is referred to the literature.^52–54^

It must be noted that the solvent plays an important role in oxidizer strength. For example, I_2_ is oxidized by UF_6_ and MoF_6_ in acetonitrile but only by UF_6_ in IF_5_ as a solvent,^5^ Xe reacts in aHF with F_2_ in the dark, see above, but not if dissolved in WF_6_.^55^ Neat NiF_3_ ^56^ does not oxidize Xe; however, in aHF, XeF_2_ or even Xe­(VI) compounds may be obtained.^57^ The same is true for K_2_[NiF_6_].^58^

The addition of soluble Lewis acids increases the oxidizer strength of F_2_ even more, with the whole system becoming more acidic. For example, NF_3_ is only oxidized by F_2_ to the NF_4_^+^ cation in aHF when a Lewis acid such as AsF_5_ or SbF_5_ is present.^59^ Neat Xe and F_2_ do not react at room temperature in the dark (the reaction is unmeasurably slow);^60^ however, in aHF as a solvent, the reaction forming XeF_2_ proceeds smoothly.^45,55,61^ To the best of our knowledge, it is still not understood why aHF promotes this reaction in the dark.

Finally, we mention that the acidity and basicity of the aHF may have an enormous influence on the outcome of the reactions.^44^

For all of the reasons above, aHF might be a suitable solvent, and RuF_6_ might be a suitable oxidizer for our task, especially since we decided to explore its chemistry.

The existence of [XeF_5_]^+^ and [Xe_2_F_11_]^+^ cations as stable species has been inferred early on because of the high electrical conductivity of solutions of XeF_6_ in aHF. The chemical equilibria are described with eqs 7–9 in the literature.^62–64^

$${XeF}_{6}+HF\overset{\begin{array}{c} aHF \end{array}}{\begin{array}{c} ⇄ \end{array}}{\left[{XeF}_{5}\right]}^{+}+{\left[{HF}_{2}\right]}^{-}$$ 7

([XeF5]F+)4+2nHF⇄aHF2[Xe2F11]++2[(HF)nF]− 8

$${\left[{Xe}_{2}F_{11}\right]}^{+}+nHF\overset{\begin{array}{c} aHF \end{array}}{\begin{array}{c} ⇄ \end{array}}2{\left[{XeF}_{5}\right]}^{+}+{\left[{\left(HF\right)}_{n}F\right]}^{-}$$ 9

As we observe the same product in the reaction of XeF_6_ and RuF_6_ without aHF as a solvent, its presence does not seem to play a role in this case. Such behavior has been observed previously: “... the presence of HF could markedly affect the kinetics of redox reactions involving higher fluorides of transition metals. However, in most reactions the nature of the final product was independent of the presence or absence of HF”.^65^

It has been noted before that pure RuF_6_ decomposes at room temperature to RuF_5_ and F_2_, with the decomposition being so slow that RuF_6_ can be kept in a nickel vessel for weeks.^35^ We essentially observed the same behavior for RuF_6_ dissolved in aHF at room temperature inside PFA or FEP vessels. However, additional decomposition does slightly occur because of moisture and oxygen diffusing through the walls of the fluoropolymer vessels, forming [O_2_]^+^ species. The formation of the compounds [XeF_5_]­[RuF_6_] and [Xe_2_F_11_][RuF_6_], from either neat or from RuF_6_ and XeF_6_ dissolved in aHF, is essentially instantaneous. We therefore assume that decomposition of RuF_6_ under formation of F_2_ and the Lewis acid RuF_5_ is not the decisive step of the reactions that we observed.

### Consideration of Redox Potentials

While electrofluorination (Simons process) is an important and established process in academic laboratories and industry,^66,67^ there seem to be no determinations of redox potentials or the establishment of a comprehensive Galvanic series for anhydrous HF. However, the behavior of some metals in aHF has been investigated.^68,69^ Also, some potentiometric measurements were carried out,^70–72^ and we know of only one recent investigation in the silver system.^73^ To the best of our knowledge, transition metal fluorides in high oxidation states have been investigated electrochemically both only once for MoF_6_ and WF_6_.^65,74^ In summary, we are not aware of redox potentials or a Galvanic series for aHF that would help us to explain the reactions we observed. Redox potentials may be calculated quantum-chemically,^75^ however, experimental data are required to anchor the results obtained from theory. We leave these tasks for others.

### Consideration of Fluoride Ion Affinities (FIAs)

FIAs have been calculated for the relevant species for the gas phase^20,76^ as well as using solvation models (COSMO) for RuF_5_ in *D*_3*h*_ symmetry.^76^ However, the local minimum for the RuF_5_ molecule is not the trigonal bipyramid, but a square pyramid. Therefore, we recalculated the FIAs of the species of interest with and without solvent model on the same level of theory. The relevant reactions and FIAs are given in eqs 10–12. For details of the quantum-chemical calculations see the Experimental Section.

$${\left[{XeF}_{5}\right]}^{+}+F^{-}\to {XeF}_{6},{FIA}_{CP}=-367\;kJ/mol,\;FIA=-662\;kJ/mol$$ 10

$${\left[{XeF}_{7}\right]}^{+}+F^{-}\to {XeF}_{8},{FIA}_{CP}=-379\;kJ/mol,\;FIA=-865\;kJ/mol$$ 11

$${RuF}_{5}+F^{-}\to {\left[{RuF}_{6}\right]}^{-}{,FIA}_{CP}=-462\;kJ/mol,\;FIA=-514\;kJ/mol$$ 12

Therefore, the reactions given in eqs 13 and 14

$${XeF}_{6}+{RuF}_{5}\to {\left[{XeF}_{5}\right]}^{+}+{\left[{RuF}_{6}\right]}^{-},\Delta H_{CP}=-95\;kJ/mol,\;\Delta H=+148\;kJ/mol$$ 13

$${XeF}_{8}+{RuF}_{5}\to {\left[{XeF}_{7}\right]}^{+}+{\left[{RuF}_{6}\right]}^{-},\Delta H_{CP}=-83\;kJ/mol,\;\Delta H=+351\;kJ/mol$$ 14

are exothermic when the quantum-chemical solvent model is considered and endothermic, when it is not.

We note that the reaction enthalpies calculated via the FIAs with solvent corrections in eqs 13 and 14 are quite similar. And, as we observe the formation of solid [XeF_5_]­[RuF_6_] from XeF_6_ and RuF_6_ in aHF as almost instantaneous, we may conclude that this could also be the case for the reaction of the putative XeF_8_ with RuF_6_, especially under formation of solid [XeF_7_]­[RuF_6_], see below.

### Estimations of the Lattice Energies

Bartlett and co-workers have provided a formula for the estimation of lattice energies, *U*_L_, which is especially helpful when complex cations, of low symmetry, are involved. Radii of cations and anions are not needed then as the cube root of the formula unit volume (FUV/Å^3^) is used, see eq 15.^55,77^

15 $$U_{L}\left(kJ/mol\right)=2329.1\cdot {\left(FUV/{Å}^{3}\right)}^{-1/3}+110.1$$

Using this formula, we obtain for [XeF_5_]­[RuF_6_], using the FUV of 188.6 Å^3^,^12^ an approximate lattice energy of

$$U_{L}\left(\left[{XeF}_{5}\right]\left[{RuF}_{6}\right]\right)=-516\;kJ/mol$$

With eq 13, overall energies of −611 kJ/mol (CP) and −368 kJ/mol are obtained. Both values would be consistent with the observed formation of the salt [XeF_5_]­[RuF_6_].

As no volume of the [XeF_7_]^+^ cation is known, we approximate its volume by using the FUV of the IF_7_ molecule, which is ca. 112.8 Å^3^.^78^ As the [XeF_7_]^+^ cation should be smaller, the lattice energy would increase. To obtain the approximate volume of the [RuF_6_]^−^ anion, we calculate the FUV of Cs­[RuF_6_] to ca. 138 Å^3^, subtract 19 Å^3^ for the volume of the Cs^+^ cation, and obtain the approximate volume of the [RuF_6_]^−^ anion of 119 Å^3^ (we are aware that atom volumes are not always additive). This leads to a FUV for the putative [XeF_7_]­[RuF_6_] of 232 Å^3^ and the lattice energy is

$$U_{L}\left(\left[{XeF}_{7}\right]\left[{RuF}_{6}\right]\right)=-489\;kJ/mol$$

Overall, the lattice energy of [XeF_7_]­[RuF_6_] would lead, together with the reaction energies in eq 14, to formation energies of −572 kJ/mol (CP) and −138 kJ/mol. This indicates that [XeF_7_]­[RuF_6_] would be a feasible compound.

### Further Thermodynamic Considerations

For XeF_8_, the formation enthalpy has been calculated to be circa −172 kJ/mol.^20^ However, it is predicted to be thermodynamically unstable with respect to F_2_ loss by ca. 93 kJ/mol at 0 K.^20^ So, at higher temperatures the driving force for decomposition should increase because of the −*T*Δ*S* term in the Gibbs enthalpy, rendering it less likely to isolate that compound. The cation [XeF_7_]^+^ is also predicted to be thermodynamically unstable with respect to the loss of F_2_ by ca. 191 kJ/mol at 0 K.^20^ However, packing these cations with suitable anions into a solid changes the surrounding completely and this may provide a stabilizing effect.

So, why does RuF_6_ fail to oxidize XeF_6_?

Well, it may be that our reaction conditions are not right, yet. Maybe our wish for a Xe­(VIII) compound is naïve, but we had and still have hope to stabilize it as an ionic solid-state compound.

Under the conditions investigated by us so far, we only observe F_2_ elimination and no evidence of any Xe­(VIII) compound.

### Characterization of [Xe_2_F_11_]­[RuF_6_]

[Xe_2_F_11_]­[RuF_6_], μ-fluorido­(bis­(pentafluoridoxenonium­(VI)) hexafluoridoruthenate­(V) crystallizes as colorless needles in the monoclinic space group *P*2_1_/*n* (No. 14, *mP*80, 14 *e* ^20^) with the lattice parameters *a* = 8.4517(3), *b* = 8.9602(3), *c* = 15.4691(5) Å, β = 95.392(1)°, *V* = 1166.27(7) Å^3^, *Z* = 4, *T* = 100 K. Further details on the crystal structure determination are given in Table 1. The compound crystallizes in the [Xe_2_F_11_]­[VF_6_] structure type, similar to [Xe_2_F_11_]­[SbF_6_].^79,80^ In addition to these two compounds, another compound with such a composition is known, [Xe_2_F_11_]­[AuF_6_].^81^ It is reported to crystallize in the orthorhombic space group *Pnma.* All these compounds share the structure motif shown in Figure 1.

**Table 1:** Selected Crystallographic Data and Details of the Structure Determination on Single Crystals of [Xe_2_F_11_]­[RuF_6_]

compound	[Xe_2_F_11_][RuF_6_]
formula	F_17_RuXe_2_
molar mass/g·mol^–1^	686.67
space group (no.)	*P*2_1_/*n* (14)
*a*/Å	8.4517(3)
*b*/Å	8.9602(3)
*c*/Å	15.4691(5)
β/°	95.392(1)
*V*/Å^3^	1166.27(7)
*Z*	4
Pearson symbol	*mP*80
ρ_calc_/g·cm^–3^	3.911
μ/mm^–1^	7.269
color	colorless
crystal morphology	needle
crystal size/mm^3^	0.052·0.064·0.184
*T*/K	100
λ/Å	0.71073 (Mo–K_α_)
no. of reflections	48,303
θ range/deg	5.26–61.06
range of Miller indices	–12 ≤ *h* ≤ 12
	–12 ≤ *k* ≤ 12
	–22 ≤ *l* ≤ 22
abs. correction	numerical
completeness of the data set	0.999
no. of unique reflections	3570
no. of parameters	182
no. of restraints	0
no. of constraints	0
*S* (all data)	1.163
*R*(*F*) (*I* ≥ 2σ(*I*), all data)	0.0176, 0.0214
*wR*(*F* ^2^) (*I* ≥ 2σ(*I*), all data)	0.0345, 0.0357
extinction coeff.	0.00037(4)

**Figure 1:**
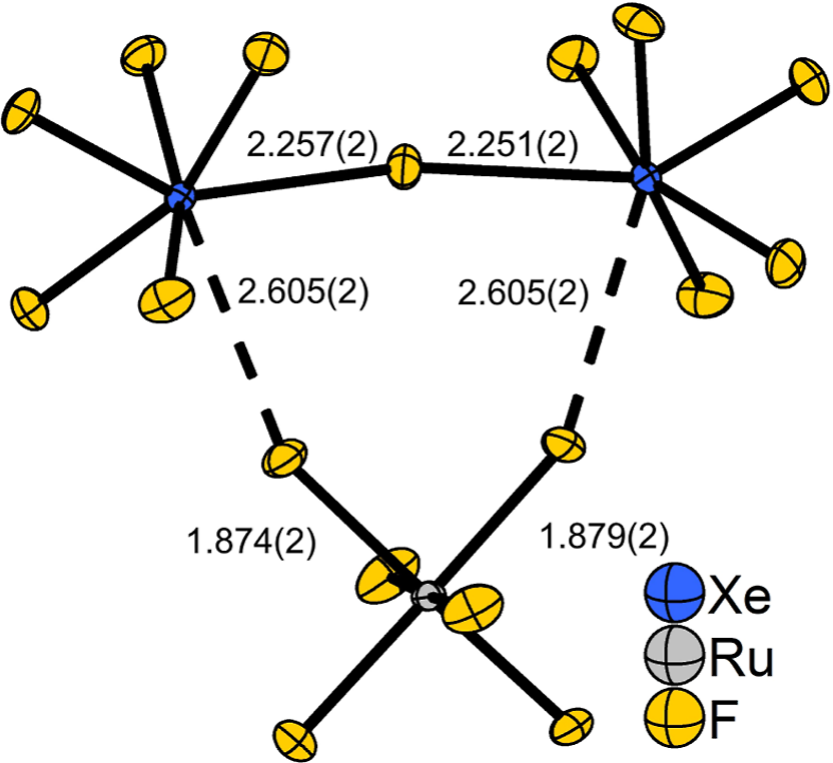
Section of the crystal structure of [Xe_2_F_11_]­[RuF_6_] showing the [Xe_2_F_11_]^+^ cation coordinated to the [RuF_6_]^−^ anion (dashed bonds). Bond lengths are given in Å. Displacement ellipsoids are shown at 70% probability level at 100 K.

In the following, the known compounds [Xe_2_F_11_]­[*M*F_6_] (*M* = Au, Sb, V) are abbreviated with the chemical symbol of the respective metal *M* atom.^79,80,82^ The [Xe_2_F_11_]^+^ cation is virtually comprised by two [XeF_5_]^+^ cations, which are bridged by a fluoride anion. In the compound [Xe_2_F_11_]­[RuF_6_] the Xe–F–Xe angle is smallest with 163.15(9)° compared to the Au compound with 169.2(2)°, the Sb compound with 166.2(2)°, and the V compound with 166.5(4)°. In the compound [Xe_2_F_11_]_2_[NiF_6_], an even smaller Xe–F–Xe angle of 140.3(6)° is observed. However, due to the different composition of the latter compound the crystal structure differs as well, which makes a direct comparison difficult.^47^ The averaged Xe−μ–F bond length of the Ru compound is 2.2542(6) Å. It lies within the same range as in the other compounds of Au with 2.23(1) Å, of Sb with 2.254(4) Å, and of V with 2.245(8) Å.

The octahedron-like [RuF_6_]^−^ anion shows Ru–F bond lengths in the range from 1.823(2) to 1.879(2) Å, which is comparable to the averaged Ru–F bond lengths in the compounds Cs­[RuF_6_] with 1.851(3) Å^37^ or [XeF_5_]­[RuF_6_] with 1.848(4) Å.^41^ Within the [RuF_6_]^−^ anion, two of its F atoms are terminally bound in the *cis*-position showing shorter bond lengths of 1.823(2) and 1.826(2) Å, while its other four F atoms act μ_2_-bridging toward the Xe atoms of different cations, as depicted in Figure 2. Both Xe atoms of the cation are coordinated by two *cis*-positioned F atoms of a [RuF_6_]^−^ anion, with Xe···F distances of both 2.605(2) Å shown as black dashed bonds in Figure 2. In the Au, Sb, and V compounds, the respective Xe···F distances are 2.64(1), 2.636(7), and 2.555(7) Å. By this linkage of cation and anion, virtual [Xe_2_F_11_]­[RuF_6_] units are formed. These are interconnected by two *trans*-positioned F atoms of the [RuF_6_]^−^ anion with Xe···F distances of 2.893(2) and 2.967(2) Å shown as red dashed bonds in Figure 2. In the Au, Sb, and V compounds, these Xe···F distances are observed at 2.95(1), 2.942(5), and 3.163(8) Å, respectively. The interconnection of virtual [Xe_2_F_11_]­[RuF_6_] units leads to the formation of one-dimensional infinite strands which are arranged parallel to the *a* axis as depicted in Figure 3. Overall, four F atoms of the [RuF_6_]^−^ anion act as bridging ligands, while the other two, *cis*-positioned, F atoms are terminally bound.

**Figure 2:**
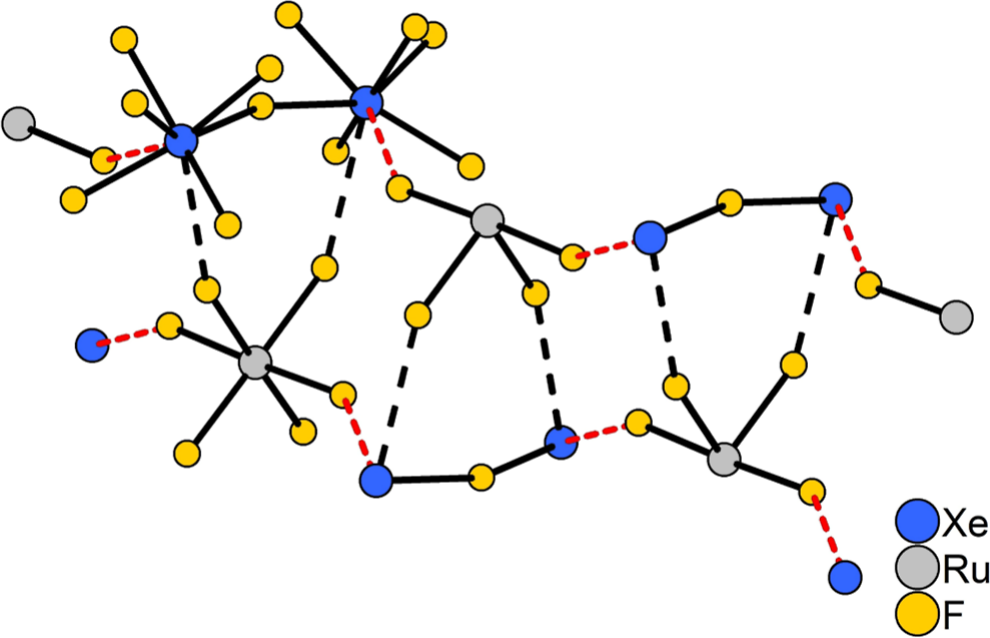
Section of the crystal structure of [Xe_2_F_11_]­[RuF_6_] showing the one-dimensional infinite strands formed by interconnection (red dashed bonds) of virtual [Xe_2_F_11_]­[RuF_6_] units (black dashed bonds). For a clearer overview only one virtual [Xe_2_F_11_]­[RuF_6_] unit is shown on the left, while then only bridging F atoms and Ru and Xe atoms are shown. All atoms are shown isotropic with arbitrary radii.

**Figure 3:**
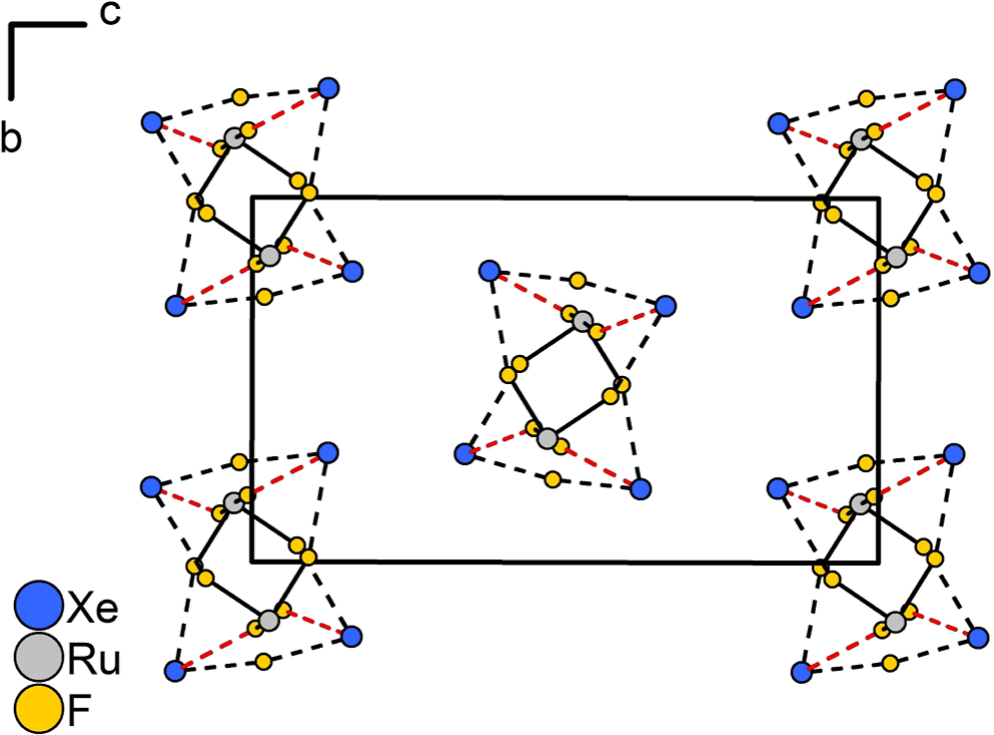
Crystal structure of [Xe_2_F_11_]­[RuF_6_]. For a clearer overview only the F atoms bridging between Xe and Ru atoms are shown. The Xe···F interactions between the [Xe_2_F_11_]^+^ cations and the [RuF_6_]^−^ anions are shown as black dashed bonds. The Xe···F interactions between the virtual [Xe_2_F_11_]­[RuF_6_] units leading to one-dimensional infinite strands parallel to the *a* axis are shown as red dashed bonds. Atoms are shown isotropic with arbitrary radii.

Vibrational spectroscopic studies in comparison to quantum chemically calculated spectra are shown in Figure 4. The calculated band positions are in good overall agreement with the measured values.

**Figure 4:**
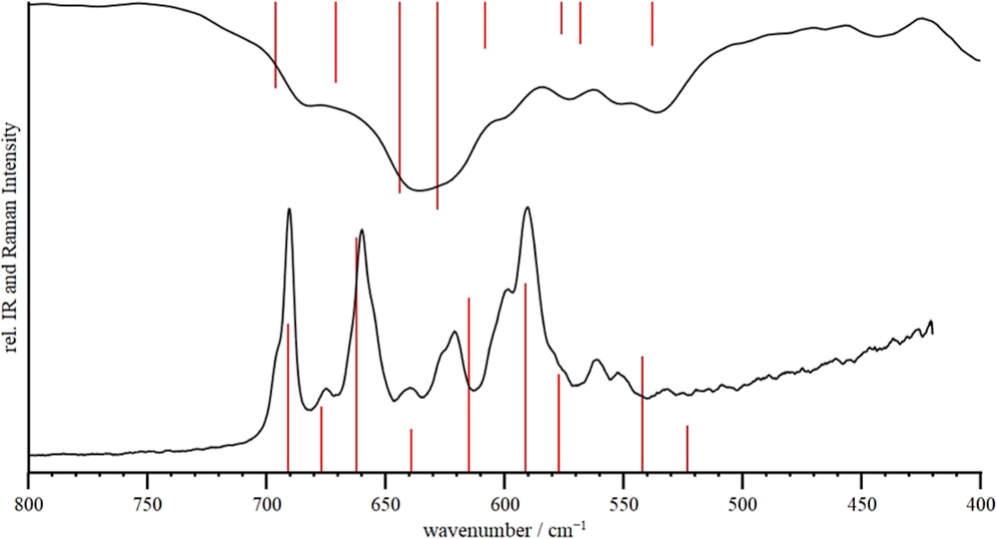
Bottom: Raman spectrum (in black) of [Xe_2_F_11_]­[RuF_6_] recorded at room temperature with an excitation laser wavelength of 532 nm through a flame-sealed quartz capillary. Top: IR spectrum (in black) of [Xe_2_F_11_]­[RuF_6_] recorded at room temperature. No bands were observed above 800 cm^–1^. The quantum-chemically calculated band positions for the solid-state compound are shown with red lines (DFT-PBE0/TZVP).

The assignment of Raman and IR bands is given in Table 2. Please refer to the Supporting Information for the full Raman and IR spectra recorded up to 4000 cm^–1^.

**Table 2:** Band Assignment of Raman and IR Spectra based on the Quantum-Chemically Calculated Ones of Solid-State [Xe_2_F_11_]­[RuF_6_]­^a^

ν(observed)/cm^–1^		ν(calculated)/cm^–1^	assignment
Raman	IR		
	696(sh)	696	F_ap_–Xe−μ-F (symmetric stretching)
690(vs)		690	Ru–F_ap_ (symmetric stretching)
674(w)		676	Ru–F_ap_ (asymmetric stretching)
	681(w)	671	F_ap_–Xe−μ-F (asymmetric stretching)
658(s)		662	Xe···μ-F–Ru−μ-F···Xe (symmetric stretching)
	636(s)	644	[Xe_2_F_11_]^+^ (various stretching vibrations)
639(w)		639	[Xe_2_F_11_]^+^ (various stretching vibrations)
	621(sh)	628	Ru−μ-F···Xe–F_ap_ (symmetric stretching)
620(m)		615	Xe···μ-F–Ru−μ-F···Xe (asymmetric stretching)
	599(sh)	608	[Xe_2_F_11_]^+^ (various stretching vibrations)
590(s)		591	[RuF_6_]^−^ (symmetric stretching)
578(sh)		577	[RuF_6_]^−^ (asymmetric stretching)
	573(w)	576	[RuF_6_]^−^ (asymmetric stretching)
	569(sh)	568	[Xe_2_F_11_]^+^ (various stretching vibrations)
560(m)		542	[RuF_6_]^−^ (asymmetric stretching)
	534(m)	537	[RuF_6_]^−^ (asymmetric stretching)
552(m)		523	[RuF_6_]^−^ (asymmetric stretching)

a The notation for band assignments is the following: vw—very weak, w—weak, m—medium, s—strong, vs—very strong, sh—shoulder, μ—bridging fluorine atom, ap—non-bridging fluorine atom. The band assignment is derived from visual inspection of the calculated normal modes using the visualization tool CRYSPLOT.^83,84^

## Conclusion

This study explored whether XeF_6_ could be oxidized by RuF_6_, either neat or dissolved in a HF solution, using different stoichiometric ratios and reaction temperatures. While a 1:2 ratio was proposed to yield the elusive Xe­(VIII) compound [XeF_7_]­[Ru_2_F_11_], all reactions led to the formation of Xe compounds in lower oxidation states instead. The main product was the colorless compound [Xe_2_F_11_]­[RuF_6_], identified by X-ray diffraction and vibrational spectroscopy. However, no evidence was obtained for the formation of the Xe­(VIII) species. We observed in the course of our investigations that reactions involving equimolar amounts or an excess of RuF_6_ resulted in the formation of F_2_ gas and other [XeF_5_]^+^-containing compounds. However, no [XeF_7_]^+^ cations were observed. The obtained compound [Xe_2_F_11_]­[RuF_6_] crystallizes isotypically to the compounds [Xe_2_F_11_]­[*M*F_6_] (*M* = Sb, V), forming one-dimensional infinite strands of interconnected cations and anions. While quantum-chemical calculations and considerations of reaction and lattice energies suggest that the [XeF_7_]^+^ cation may be stabilized as a solid compound, we did not observe its formation under our reaction conditions, as its decomposition may be too fast. In the future, we will investigate the oxidative power of solutions of Ag­(III) and Ni­(IV).

## Experimental Section

### General

All operations were performed in a Monel metal Schlenk line, which was passivated with fluorine and ClF_3_ at various temperatures and pressures before use. Ru (99.8%) was purchased from MaTeck, and Xe (4.0) was purchased from Linde. RuF_6_^51^ and XeF_6_^12^ were made according to literature procedures and stored in vials made out of Monel.

Reaction vials were made out of perfluoroalkoxy alkanes PFA and sealed with bellow valves made out of stainless steel. The vessels were baked out in vacuum (∼10^–3^ mbar) at ca. 423 K for several hours and then filled up to a pressure of 2 bar with F_2_ and heated to ca. 373 K for several hours to saturate the polymer with fluorine.^85^

**CAUTION!** Fluorine and fluorides as those presented in this paper must be handled with appropriate protective gear with ready access to proper emergency treatment procedures in the event of contact. The aforementioned are potent oxidative fluorinators that are only stable under the rigorously anhydrous conditions employed in the experimental procedures outlined in the Experimental Section. They react vigorously and explosively upon hydrolysis or contact with organic materials. The utmost precautions must be taken when disposing of these materials and their derivatives.

### Preparation of [Xe_2_F_11_]­[RuF_6_]

A PFA reaction vessel was cooled to 77 K, 30 mg of RuF_6_ (0.14 mmol), an excess of XeF_6_ (100 mg, 0.41 mmol), and 0.3 mL of aHF were condensed. The reaction mixture was then allowed to warm to room temperature. The excess of XeF_6_ and aHF was then removed under a reduced pressure. Colorless single crystals were obtained by dissolving the remaining solid in HF and slowly cooling to 243 K.

### Preparation of [XeF_5_]­[RuF_6_]

A PFA reaction vessel was cooled to 77 K, 100 mg of RuF_6_ (0.46 mmol), 120 mg of XeF_6_ (0.49 mmol), and 0.3 mL of aHF were condensed. The reaction mixture was then allowed to warm to room temperature. The aHF was then removed under reduced pressure.

### Single-Crystal X-Ray Diffraction

Single crystals were selected under predried perfluorinated oil (LS 230) at 196 K and mounted using a MiTeGen loop. Intensity data of a suitable crystal were recorded with a D8 Quest diffractometer (Bruker). The diffractometer was operated with Mo–K_α_ radiation (0.71073 Å, multi layered optics) and equipped with a PHOTON III C14 detector. Evaluation, integration, and reduction of the diffraction data were carried out using the APEX5 software suite.^86^ The diffraction data were corrected for absorption using the correction method of SADABS within the APEX5 software suite. The structures were solved with dual-space methods (SHELXT),^87^ and refined against *F* ^2^ (SHELXL) within the Shelxle suite.^88,89^

Atoms were refined with anisotropic displacement parameters. Representations of the crystal structures were created with the Diamond software.^90^ Deposition number of [Xe_2_F_11_]­[RuF_6_]: 2477117. These data are provided free of charge by the joint Cambridge Crystallographic Data Centre and Fachinformationszentrum Karlsruhe Access Structures service.

### Powder X-ray Diffraction

The samples were filled into a flame-dried quartz capillary with a diameter of 0.3 mm. The powder X-ray pattern was recorded with a StadiMP diffractometer (Stoe & Cie) in Debye–Scherrer geometry. The diffractometer was operated with Cu–K_α1_ radiation (1.5406 Å, germanium monochromator) and equipped with a MYTHEN 1 K detector.

### Infrared Spectroscopy

The IR spectrum was recorded on a Bruker alpha FT-IR spectrometer using the ATR Diamond module with a resolution of 4 cm^–1^. The spectrometer was located inside a glovebox (MBraun) under an argon atmosphere. The spectra were processed with the OPUS software package.^91^

### Raman Spectroscopy

The Raman spectra were measured at room temperature with a Monovista CRS + confocal Raman microscope (spectroscopy and Imaging GmbH) using a solid-state laser and a 300 grooves/mm grating.

### Computational Details

Periodic quantum-chemical calculations were performed using the CRYSTAL23 software suite.^92^ All calculations employed the DFT-PBE0 hybrid density functional method (PBE combined with 25% exact Hartree–Fock exchange).^93,94^ Triple-ζ valence plus polarization (TZVP) Gaussian-type basis sets were used for all atoms. The Xe atom basis set has been derived from def2-TZVP and is included in the Supporting Information. F and Ru atom basis sets have been previously reported in the literature.^95,96^

The crystal structure was fully optimized under the symmetry constraints of its space group in the standard setting. We carried out spin-polarized calculations as the Ru­(V) species possesses three unpaired *d*-electrons. The electronic structure calculations were performed assuming a ferromagnetic ordering of the Ru centers. For this magnetic configuration, a local magnetic moment of 2.30 μ_B_ for the Ru atoms was obtained. Furthermore, the calculations yielded a band gap of 5.31 eV. Reciprocal space sampling was performed using a 4 × 4 × 2 Monkhorst–Pack-type *k*-point grid.^97^ Tight tolerances (TOLINTEG = 8, 8, 8, 8, 16) were applied for the evaluation of Coulomb and exchange integrals. Default CRYSTAL23 optimization criteria and DFT integration grids were used throughout. The optimized lattice parameters deviated by less than 2.7% from those determined via single-crystal X-ray diffraction. The optimized structure was confirmed to be a true local minimum through a harmonic frequency calculation.^98,99^

Raman intensities were calculated with the schemes implemented in CRYSTAL^100^ using the same conditions as in the experimental setup (*T* = 273.15 K and λ = 532 nm). The band assignments were carried out by visual inspection of the normal modes with the CRYSPLOT online tool.^84^

FIAs have been calculated with Orca 6.1.1 ^101^ according to a slightly modified approach as presented in the literature.^76^ The respective molecular structures (COF_2_, [COF_3_]^−^, RuF_5_, [RuF_6_]^−^, [XeF_5_]^+^, XeF_6_, [XeF_7_]^+^, and XeF_8_) have been optimized by using the RIJK-PW6B95­(D3BJ)-ATM/def2-QZVPP level of theory.^102–109^ A polarizable continuum model (CPCM, ε_HF_ = 84) has been used throughout all the structure optimizations to account for the solvent influence on geometrical and electronical data of all calculated species. Two-sided numerical frequency calculations proved the structures as minima on the potential energy surface and provided the necessary data for the thermochemical calculations. An effective 28-electron core potential was used for Ru^110^ and Xe.^111^

Subsequent CCSD­(T)/def2-QZVPP calculations with^112^ or without implicit solvent contribution (CPCM, ε_HF_ = 84) provided the electronic energies needed for the FIA calculations, which have been carried out exactly as published in the literature.^76^

## Supplementary Material



## Notes

Crystallographic Details for [Xe_2_F_11_]­[RuF_6_], powder X-ray diffraction pattern and vibrational spectra of [Xe_2_F_11_]­[RuF_6_], powder X-ray diffraction pattern and Raman spectrum of [XeF_5_]­[RuF_6_], and computational details (PDF)
