# Supplementary material for: Failed Attempts at Oxidation of XeF6: Synthesis and Characterization of [Xe2F11][RuF6]
Source: Inorg Chem. 2026 Jun 24;65(26):15126–35. doi: 10.1021/acs.inorgchem.6c02072 (PMC13343514; doi:10.1021/acs.inorgchem.6c02072)
Supplement: Supplementary file 1 [file ic6c02072_si_001.pdf]

Supporting Information

Failed Attempts at Oxidation of XeF_6: Synthesis and Characterization of $[Xe_2F_{11}][RuF_6]$

Björn N. Koch[a], Giuliana Hoß[a], Gregor Schnakenburg[a], Antti J. Karttunen[b], and Florian Kraus[a,*]

[a] M. Sc. Björn N. Koch, M. Sc. Giuliana Hoß, Dr. Gregor Schnakenburg, Prof. Dr. Florian Kraus, Fluorchemie, Institut für Anorganische Chemie, Universität Bonn, Gerhard-Domagk-Strasse 1, 53121 Bonn, https://www.chemie.uni-bonn.de/kraus/de, fkraus@uni-bonn.de

[b] Prof. Dr. Antti J. Karttunen, Aalto University, Department of Chemistry and Materials Science, Kemistintie 1, Aalto, FI 00076

Table of Contents

Characterization of $[Xe_2F_{11}][RuF_6]$ 2

Structure Determination on single crystals of $[Xe_2F_{11}][RuF_6]$ and powder X-ray diffraction pattern 2

Vibrational spectra of $[Xe_2F_{11}][RuF_6]$ 4

Characterization of $[XeF_5][RuF_6]$ 5

Powder X-ray pattern of $[XeF_5][RuF_6]$ 5

Raman spectrum of $[XeF_5][RuF_6]$ 6

Computational Details 7

FIA calculations 9

References 11

Characterization of $[Xe_2F_{11}][RuF_6]$

Structure Determination on single crystals of $[Xe_2F_{11}][RuF_6]$ and powder X-ray diffraction pattern

Table S1. Wyckoff symbols, site symmetries, atomic coordinates, and isotropic displacement parameters of $[Xe_2F_{11}][RuF_6]$.

Atom	Site	Site sym.	*x*	*y*	*z*	$U_{eq/iso}$ / Å
Xe1	*4e*	1	0.74900(2)	0.70385(2)	0.33985(2)	0.74900(2)
Xe2	*4e*	1	0.72333(2)	0.79878(2)	0.62119(2)	0.72333(2)
Ru1	*4e*	1	0.74902(3)	0.33875(2)	0.52816(2)	0.74902(3)
F1	*4e*	1	0.7676(2)	0.77323(19)	0.48032(11)	0.7676(2)
F2	*4e*	1	0.7505(2)	0.45516(19)	0.42717(11)	0.7505(2)
F3	*4e*	1	0.7150(2)	0.51265(19)	0.59114(11)	0.7150(2)
F4	*4e*	1	0.9649(2)	0.3679(2)	0.55032(13)	0.9649(2)
F5	*4e*	1	0.5303(2)	0.3176(2)	0.50674(12)	0.5303(2)
F6	*4e*	1	0.7469(2)	0.22918(19)	0.62759(11)	0.7469(2)
F7	*4e*	1	0.7803(3)	0.1715(2)	0.46479(12)	0.7803(3)
F8	*4e*	1	0.7196(2)	0.7631(2)	0.22675(10)	0.7196(2)
F9	*4e*	1	0.5754(2)	0.5848(2)	0.30037(11)	0.5754(2)
F10	*4e*	1	0.8863(2)	0.5749(2)	0.28736(11)	0.8863(2)
F11	*4e*	1	0.9010(2)	0.85246(19)	0.33931(11)	0.9010(2)
F12	*4e*	1	0.6005(2)	0.8535(2)	0.34665(11)	0.6005(2)
F13	*4e*	1	0.7215(2)	0.9238(2)	0.71363(11)	0.7215(2)
F14	*4e*	1	0.5516(2)	0.7226(2)	0.67588(12)	0.5516(2)
F15	*4e*	1	0.8660(2)	0.6991(2)	0.70050(11)	0.8660(2)
F16	*4e*	1	0.8939(2)	0.9201(2)	0.60449(11)	0.8939(2)
F17	*4e*	1	0.5908(2)	0.94617(19)	0.57297(11)	0.5908(2)

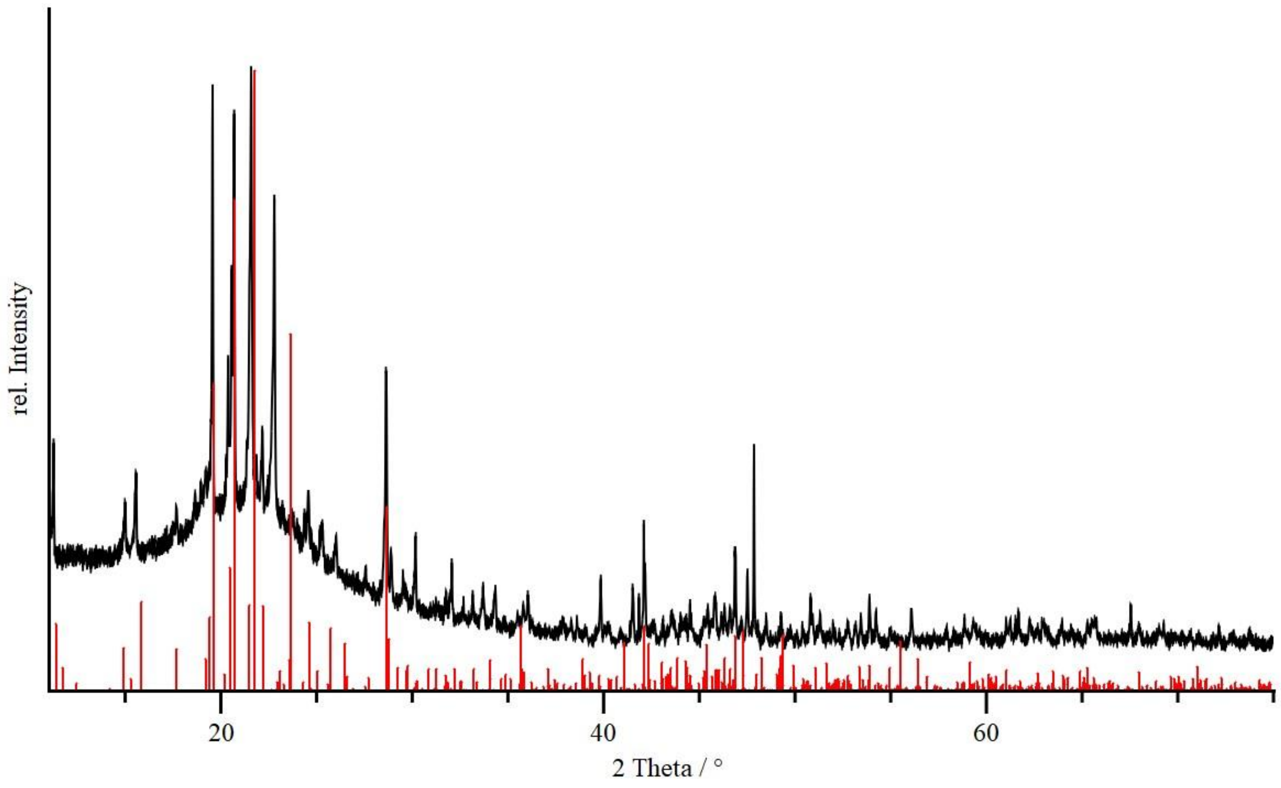

Figure S1. Powder X-ray diffraction pattern of $[Xe_2F_{11}][RuF_6]$ in black, recorded in a quartz capillary with a diameter of 0.3 mm at room temperature. The calculated reflection positions and intensities, based on the single crystal structure determination at 100 K, are indicated by vertical bars in red.

Vibrational spectra of $[Xe_2F_{11}][RuF_6]$

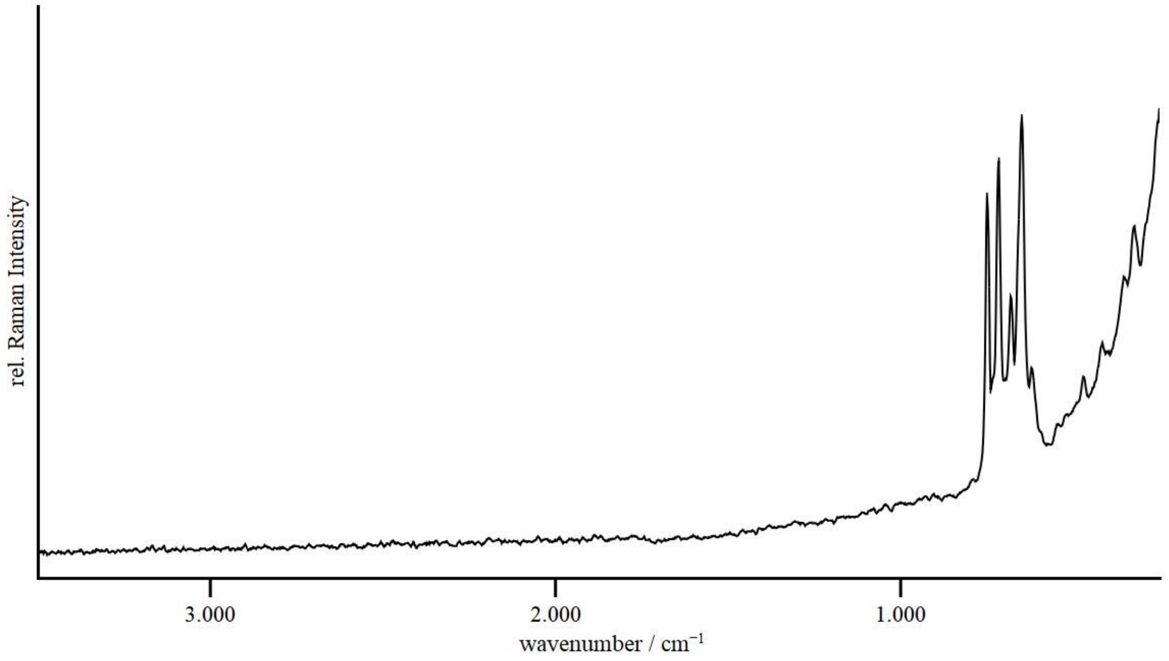

Figure S2. Raman spectrum of $[Xe_2F_{11}][RuF_6]$ recorded in a quartz capillary with a diameter of 0.3 mm at room temperature with an excitation laser wavelength of 532 nm.

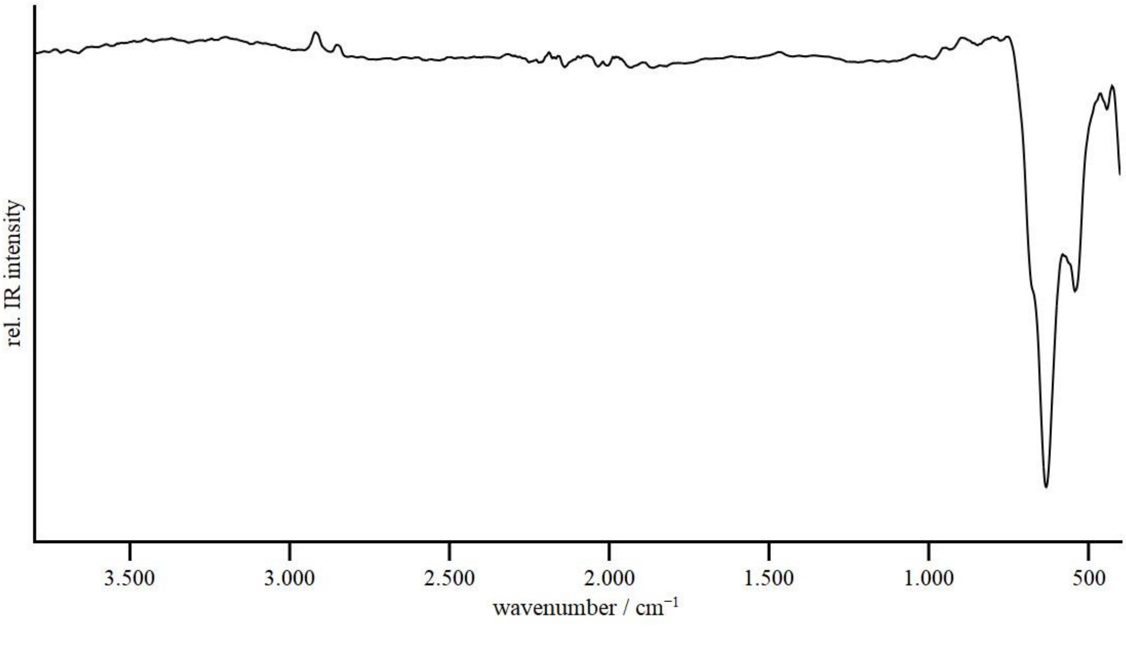

Figure S3. Infrared spectrum of $[Xe_2F_{11}][RuF_6]$ recorded at room temperature.

Characterization of [XeF_5][RuF_6]

Powder X-ray pattern of [XeF_5][RuF_6]

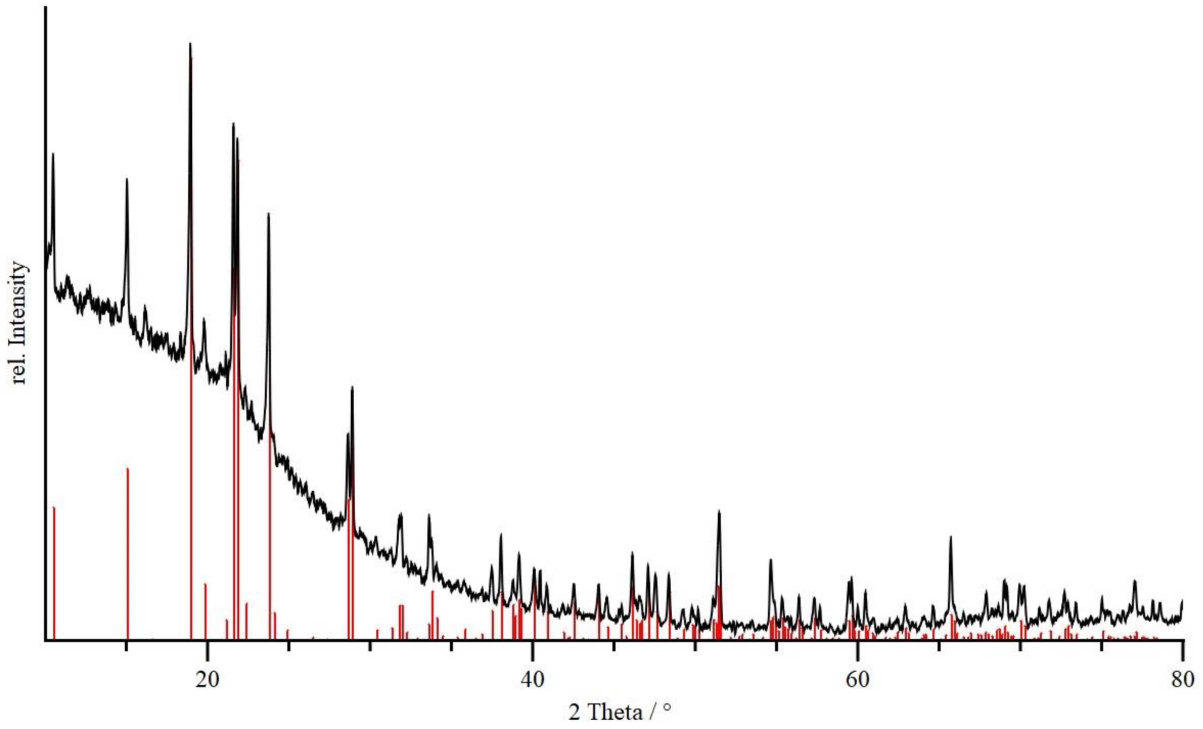

Figure S4. Powder X-ray diffraction pattern of [XeF_5][RuF_6] in black, in a quartz capillary with a diameter of 0.3 mm at room temperature. The calculated reflection positions and intensities, based on the single crystal structure determination at 100 K, are indicated by vertical bars in red.[1,2]

Raman spectrum of $[XeF_5][RuF_6]$

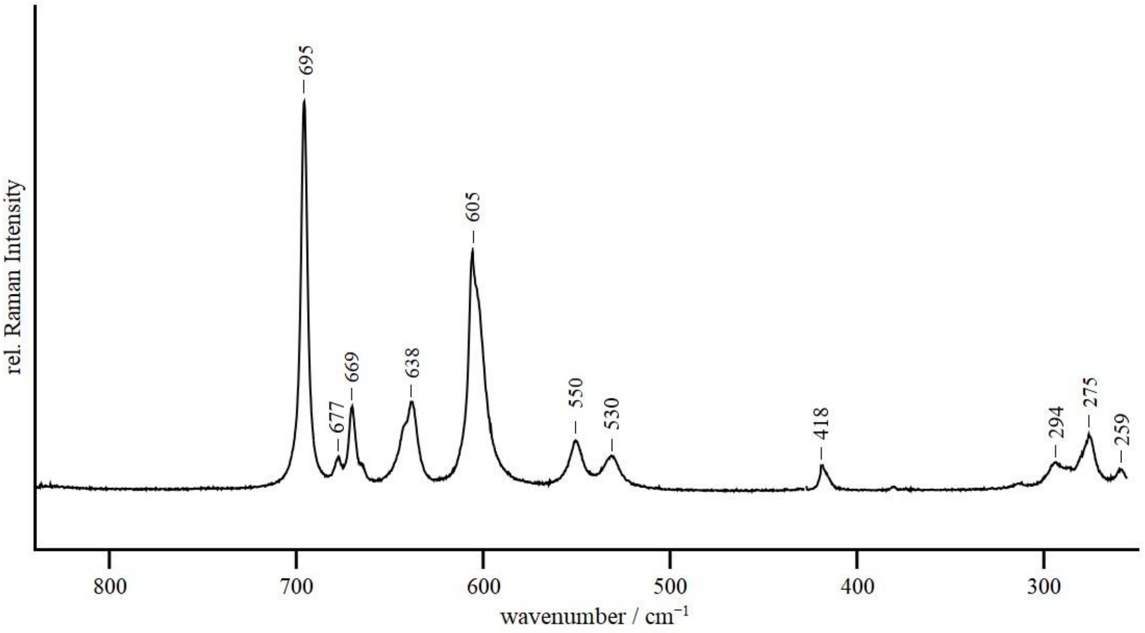

Figure S5. Raman spectrum of $[XeF_5][RuF_6]$ recorded in a quartz capillary with a diameter of 0.3 mm at room temperature with an excitation laser wavelength of 532 nm.

Table S2. Band assignment for the recorded Raman spectra of $[XeF_5][RuF_6]$. The notation for band assignments is the following: vw – very weak, w – weak, m – medium, s – strong, vs – very strong,. The normal modes of vibration are given of a tetragonal-pyramidal molecule for $[XeF_5]^+$ and for a octahedral molecule for $[RuF_6]^-$. The band assignment for $[XeF_5][RuF_6]$ is derived from literature.[3] The splitting of the bands T_{1g} and E_g for $[RuF_6]^-$ could be explained by the C_{4v} distortion of the anion, which also occurs in the literature.

ν(observed) / cm^{-1}		Assignment
$[XeF_5][RuF_6]$	$[XeF_5][RuF_6]$[3]	
695(vs)	696	$[RuF_6]^-$ ν_1 (A_{1g})
677(vw)	677	$[XeF_5]^+$ ν_7 (E)
669(m)	670	$[XeF_5]^+$ ν_1 (A_1)
638(m)	638	$[RuF_6]^-$ ν_3 (T_{2u})
605(s)	606	$[XeF_5]^+$ ν_2 (A_1)
550(w)	549	$[RuF_6]^-$ ν_2 (E_g)
530(w)	530	
418(vw)	416	$[XeF_5]^+$ ν_8 (E)
294(vw)	293	$[RuF_6]^-$ ν_4 (T_{1u})
275(w)	275	$[RuF_6]^-$ ν_5 (T_{2g})
259(w)	258	

Computational Details

For the Xe atom basis set the def2-TZVP basis set[4] was used as the starting point. The 1*s*, 2*s*, 2*p*, 3*s*, 3*p*, and 3*d* core electrons were described using a 28-electron scalar-relativistic pseudopotential from Stuttgart-Cologne.[5] The def2-TZVP basis set was then modified by fixing the exponents of the outermost *s* and *p* functions to 0.14, and reoptimizing the exponents of the remaining *s* and *p* functions in the valence space for the Xe atom in its ground state. These optimizations were performed using the TURBOMOLE program package.[6] To enhance computational efficiency in the CRYSTAL code, the outermost *s* and *p* functions were combined into a single *sp*-type function. The overall energy loss compared to the unmodified def2-TZVP basis set is 3.9 mH. The set of *d* polarization functions was adjusted by increasing the exponent of the outermost d function from 0.18 to 0.29. Additionally, the set of *f* polarization functions was reduced by removing the steepest function, with an exponent of 2.5. The full basis set, in CRYSTAL input format, is provided below:

Xe_tzvp

```
254 14
INPUT
26. 0 3 4 6 4 0
40.005184   49.997962 0
17.812214  281.013303 0
9.304150    61.538255 0
15.701772   67.439142 0
15.258608  134.874711 0
9.292184    14.663300 0
8.559003    29.354730 0
15.185600   35.436908 0
14.284500   53.195772 0
7.121889     9.046232 0
6.991963    13.223681 0
0.623946     0.084853 0
0.647284     0.044155 0
20.881557  -23.089295 0
20.783443  -30.074475 0
5.253389   -0.288227 0
5.361188   -0.386924 0
0 0 5 2.0 1.0
  6420.2481656      0.25092173886E-03
  983.54530664      0.16251948178E-02
  219.43881364      0.46037106451E-02
  23.012587807     -0.14698707182
  18.048324490      0.57524870348
0 0 2 2.0 1.0
  11.758861011      0.66038420156
  6.2520248341      0.38470524721
0 0 1 0.0 1.0
 2.6220335498       1.0000000000
0 0 1 0.0 1.0
 1.2899444222       1.0000000000
0 0 1 0.0 1.0
 0.36032717540       1.0000000000
0 1 1 0.0 1.0
 0.14                1.0 1.0
0 2 3 6.0 1.0
  193.81432545      0.95394802497E-03
  21.725228086      0.57393353332E-01
  9.8891605641     -0.27974266640
```

```
 0 2 4 6.0 1.0
   14.439417376     -0.50950157807E-01
   4.1678246384      0.36669211800
   2.3446871693      0.72619456861
   1.3581063447      0.35555871740
 0 2 1 0.0 1.0
   0.83137850050     1.0000000000
 0 2 1 0.0 1.0
   0.39977941862     1.0000000000
 0 3 6 10.0 1.0
   135.60300038      0.81873543290E-03
   38.727062692      0.60897654151E-02
   15.377328089     -0.92782985596E-02
   5.2602537686      0.22890785589
   2.6590627424      0.44434407051
   1.2938205124      0.36029482992
 0 3 1 0.0 1.0
  0.58050830139      1.0000000000
 0 3 1 0.0 1.0
  0.290254150695      1.0000000000
 0 4 1 0.0 1.0
  0.49652793000       1.0000000000
```

The crystal structure of the compound $[Xe_2F_{11}][RuF_6]$ was transformed into the standard setting $P2_1/c$ (14) using the Bilbao Crystallographic server[7–9] and was optimized with the following input lattice parameters: $a = 8.4517$, $b = 8.9602$ and $c = 16.91557397$ Å and $\beta = 114.4359483°$. In the following section, atomic positions and lattice parameters of the optimized structure are given in CIF format created from the CRYSTAL output using the FINDSYM program.[10]

```
data_findsym-output
_audit_creation_method FINDSYM

_cell_length_a    8.6824947700
_cell_length_b    9.1357121500
_cell_length_c    17.2310234011
_cell_angle_alpha 90.0000000000
_cell_angle_beta  114.7376117688
_cell_angle_gamma 90.0000000000
_cell_volume      1241.3538898909

_symmetry_space_group_name_H-M "P 1 21/c 1"
_symmetry_Int_Tables_number 14
_space_group.reference_setting '014:-P 2ybc'
_space_group.transform_Pp_abc a,b,c;0,0,0

loop_
_space_group_symop_id
_space_group_symop_operation_xyz
1 x,y,z
2 -x,y+1/2,-z+1/2
3 -x,-y,-z
4 x,-y+1/2,z+1/2

loop_
_atom_site_label
_atom_site_type_symbol
_atom_site_symmetry_multiplicity
_atom_site_Wyckoff_label
_atom_site_fract_x
_atom_site_fract_y
_atom_site_fract_z
_atom_site_occupancy
_atom_site_fract_symmform
Xe1 Xe   4 e -0.09008 0.79382  0.15675  1.00000 Dx,Dy,Dz
```

```
Xe2 Xe   4 e 0.60413  0.70785  0.87791  1.00000 Dx,Dy,Dz
Ru1 Ru   4 e 0.72273  0.15675  -0.02557 1.00000 Dx,Dy,Dz
F1  F    4 e 0.77986  0.73584  0.01693  1.00000 Dx,Dy,Dz
F2  F    4 e -0.00140 0.72614  0.26830  1.00000 Dx,Dy,Dz
F3  F    4 e 0.05323  0.64557  0.15075  1.00000 Dx,Dy,Dz
F4  F    4 e 0.75289  0.64782  0.14875  1.00000 Dx,Dy,Dz
F5  F    4 e 0.10147  -0.08270 0.20925  1.00000 Dx,Dy,Dz
F6  F    4 e 0.78749  -0.08919 0.20212  1.00000 Dx,Dy,Dz
F7  F    4 e 0.50922  0.58032  0.78683  1.00000 Dx,Dy,Dz
F8  F    4 e 0.78778  0.58333  0.89794  1.00000 Dx,Dy,Dz
F9  F    4 e 0.51614  0.56701  -0.07295 1.00000 Dx,Dy,Dz
F10 F    4 e 0.67091  0.80048  0.79867  1.00000 Dx,Dy,Dz
F11 F    4 e 0.38236  0.78615  0.82191  1.00000 Dx,Dy,Dz
F12 F    4 e 0.63241  -0.01593 -0.09209 1.00000 Dx,Dy,Dz
F13 F    4 e 0.82557  0.03577  0.07359  1.00000 Dx,Dy,Dz
F14 F    4 e 0.52672  0.16960  -0.00548 1.00000 Dx,Dy,Dz
F15 F    4 e -0.08231 0.13622  -0.04570 1.00000 Dx,Dy,Dz
F16 F    4 e 0.62204  0.26969  0.87705  1.00000 Dx,Dy,Dz
F17 F    4 e 0.81189  0.32064  0.04090  1.00000 Dx,Dy,Dz

# end of cif
```

FIA calculations

The charges, spin-states, RIJK-PW6B95(D3BJ)-ATM/def2-QZVPP optimized energies, and cartesian coordinates of the calculated species are given below.

COF_2

```
0 1
E -313.518175970110
  C          -8.77316783592410      0.00000000009960     -8.17483515473551
  O          -7.76155709676266     -0.00000000003929     -7.59058022276967
  F          -9.96292458063430     -0.00000000003017     -7.64172131810380
  F          -8.90580548667894     -0.00000000003015     -9.47189630439100
```

COF_3^-

```
-1 1
E -413.690923743316
  C          -9.56339258097997     -0.06481618048087     -6.94374290610688
  O          -8.98951099744330     -1.05952641327289     -6.53723894372605
  F          -9.65218638598734      0.08751575252093     -8.34122167062410
  F         -10.91125397411363      0.08757189104406     -6.56073825572211
  F          -9.02226406147574      1.17787395018876     -6.56038222382084
```

RuF_5

```
0 4
E -594.936050471017
  Ru          0.15385070551625      0.00010201960020      0.00000000254958
  F           0.36943269842881     -1.81490956524098     -0.00000000474047
  F           0.36824611428670      0.00022438592439     -1.81514810099529
  F           0.36717071138845      1.81537322693273     -0.00000000320404
  F          -1.62694625208172     -0.00101445285310     -0.00000000073482
  F           0.36824612246150      0.00022438563675      1.81514810712503
```

RuF_6^-

```
-1 4
E -695.201829099975
  Ru         -9.85000447828261      0.00000210881548    -11.02499698553421
  F         -11.70587189392354     -0.00000153805958    -11.02500147011645
  F          -9.85000176163610      0.00000169096837    -12.88087990610098
  F          -9.84999863297257     -0.00000152622366     -9.16912362553166
  F          -7.99412283459932      0.00000171423907    -11.02499826692267
  F          -9.84999863652198      1.85587540347218    -11.02500145500873
  F          -9.85000176206383     -1.85587785321185    -11.02499829078523
```

XeF_5^+

```
1 1
E -828.899807141816
  Xe         -9.85003437640567     -0.01539750931117    -11.02502179135791
  F         -11.66602309818357      0.22682667585448    -11.02498489434373
  F          -9.85005759319959      0.22683647240959    -12.84100804494830
  F          -9.85000379306770      0.22666864981812     -9.20901200778936
  F          -8.03400403829917      0.22653327769456    -11.02503871890666
  F          -9.84987710084423      1.77853243353443    -11.02493454265397
```

XeF_6
0 1
E -929.133399185702

Xe	-9.85001617419480	-0.00023938113322	-11.02518877589680
F	-11.77078081565733	0.00029396508668	-11.02477349697179
F	-9.84998034098770	0.00029442282811	-12.94606562049041
F	-9.84998038593773	0.00029351788088	-9.10464877608644
F	-7.92928152873833	0.00029388905563	-11.02477354959512
F	-9.84998042144701	1.92027380357661	-11.02477546762500
F	-9.84998033303700	-1.92121021729470	-11.02477431333441

XeF_7^+
1 1
E -1028.667465439011

F	-0.00292403930312	0.00000653845599	0.21809747573357
Xe	-0.00280736961056	0.00000680664332	1.99999999824274
F	1.84106256618726	-0.00000712988294	2.00000000303669
F	0.57272244108878	1.74791982383321	1.99999999927931
F	-1.48896468725119	1.08333694605063	2.00000000215806
F	-1.48898437376439	-1.08329691271231	2.00000000400511
F	0.57269250438543	-1.74791460968611	1.99999999932504
F	-0.00292404173221	0.00000653729820	3.78190251821947

XeF_8
0 1
E -1128.911084116427

Xe	-0.00000000001124	0.00000000053818	0.00000000116095
F	0.59904024028937	-1.46199585254252	-1.01255464772540
F	1.46199585175066	0.59904024260330	-1.01255464787480
F	-0.59904024202942	1.46199584817550	-1.01255464658721
F	-1.46199585588436	-0.59904023933784	-1.01255464514314
F	0.59904024493820	1.46199585197313	1.01255464907842
F	-1.46199585425056	0.59904024610909	1.01255464182697
F	1.46199585186439	-0.59904023964237	1.01255465114038
F	-0.59904023666703	-1.46199585787648	1.01255464412383

References

(1) Bartlett, N.; Gennis, M.; Gibler, D. D.; Morrell, B. K.; Zalkin, A. Crystal Structures of $[XeF^+][RuF_6^-]$ and $[XeF_5^+][RuF_6^-]$. *Inorg. Chem.* **1973**, *12* (8), 1717–1721. https://doi.org/10.1021/ic50126a002.

(2) Mazej, Z.; Goreshnik, E. Crystal Structures of Xenon(VI) Salts: $XeF_5Ni(AsF_6)_3$, XeF_5AF_6 (A = Nb, Ta, Ru, Rh, Ir, Pt, Au), and $XeF_5A_2F_{11}$ (A = Nb, Ta). *Molecules* **2023**, *28* (8), 3370. https://doi.org/10.3390/molecules28083370.

(3) Adams, C. J.; Bartlett, N. Tautomerism in Xenon Hexafluoride: An Investigation of Xenon Hexafluoride and Its Complexes by Raman Spectroscopy. *Isr. J. Chem.* **1978**, *17* (1–2), 114–125. https://doi.org/10.1002/ijch.197800015.

(4) Weigend, F.; Häser, M.; Patzelt, H.; Ahlrichs, R. RI-MP2: Optimized Auxiliary Basis Sets and Demonstration of Efficiency. *Chem. Phys. Lett.* **1998**, *294* (1–3), 143–152. https://doi.org/10.1016/S0009-2614(98)00862-8.

(5) Metz, B.; Stoll, H.; Dolg, M. Small-Core Multiconfiguration-Dirac–Hartree–Fock-Adjusted Pseudopotentials for Post-d Main Group Elements: Application to PbH and PbO. *J. Chem. Phys.* **2000**, *113* (7), 2563–2569. https://doi.org/10.1063/1.1305880.

(6) Ahlrichs, R.; Bär, M.; Häser, M.; Horn, H.; Kölmel, C. Electronic Structure Calculations on Workstation Computers: The Program System Turbomole. *Chem. Phys. Lett.* **1989**, *162* (3), 165–169. https://doi.org/10.1016/0009-2614(89)85118-8.

(7) Aroyo, M. I.; Perez-Mato, J. M.; Orobengoa, D.; Tasci, E. Crystallography Online: Bilbao Crystallographic Server. *Bulg. Chem. Commun.* **2011**, *43* (2), 183–197.

(8) Aroyo, M. I.; Perez-Mato, J. M.; Capillas, C.; Kroumova, E.; Ivantchev, S.; Madariaga, G.; Kirov, A.; Wondratschek, H. Bilbao Crystallographic Server: I. Databases and Crystallographic Computing Programs. *Z. Kristallogr. - Cryst. Mater.* **2006**, *221* (1), 15–27. https://doi.org/10.1524/zkri.2006.221.1.15.

(9) Aroyo, M. I.; Kirov, A.; Capillas, C.; Perez-Mato, J. M.; Wondratschek, H. Bilbao Crystallographic Server. II. Representations of Crystallographic Point Groups and Space Groups. *Acta Crystallogr., Sect. A: Found. Crystallogr.* **2006**, *62* (2), 115–128. https://doi.org/10.1107/S0108767305040286.

(10) Stokes, H. T.; Hatch, D. M. *FINDSYM*: Program for Identifying the Space-Group Symmetry of a Crystal. *J. Appl. Crystallogr.* **2005**, *38* (1), 237–238. https://doi.org/10.1107/S0021889804031528.
